# Predictive Value of Monocyte-To-Lymphocyte Ratio in Differentiating Heart Failure with Reduced Ejection Fraction in Patients with Severe Aortic Stenosis—A Retrospective Analysis

**DOI:** 10.3390/jcm13206249

**Published:** 2024-10-19

**Authors:** Anna Olasińska-Wiśniewska, Tomasz Urbanowicz, Bartłomiej Perek, Marcin Misterski, Kajetan Grodecki, Marek Grygier, Ewa Straburzyńska-Migaj, Marek Jemielity

**Affiliations:** 1 Department of Cardiac Surgery and Transplantology, Poznan University of Medical Sciences, 61-848 Poznan, Poland; turbanowicz@ump.edu.pl (T.U.); bperek@ump.edu.pl (B.P.); mister@poczta.onet.pl (M.M.); mjemielity@poczta.onet.pl (M.J.); 2 First Department of Cardiology, Medical University of Warsaw, 02-097 Warsaw, Poland; kajetan.grodecki@gmail.com; 3 First Department of Cardiology, Poznan University of Medical Sciences, 61-848 Poznan, Poland; mgrygier@wp.pl (M.G.); ewa.straburzynska-migaj@skpp.edu.pl (E.S.-M.)

**Keywords:** aortic stenosis, HFrEF, MLR

## Abstract

**Background/Objectives**: Advanced calcific aortic stenosis, with or without coronary artery disease [CAD], may lead to severe systolic dysfunction. The aim of the study was to reveal clinical and laboratory parameters that may differentiate patients with severe aortic stenosis with and without systolic dysfunction. **Methods**: A retrospective, single-center study included all consecutive patients diagnosed with severe aortic stenosis with overt heart failure. Patients with hematological and neoplastic diseases were excluded. Demographic, clinical and laboratory data were analysed. Neutrophil-to-lymphocyte [NLR], monocyte-to-lymphocyte [MLR], and platelet-to-lymphocyte [PLR] ratios were calculated. The study group was divided based on left ventricular ejection fraction [LVEF]. **Results**: The final study population comprised 301 patients [133 males [44%]; median [Q1–3] age of 80 [75–83] years]. Co-morbidities included CAD [48.8%], arterial hypertension [75.4%], diabetes mellitus [n = 124, 41.2%], atrial fibrillation [39.2%], chronic kidney disease [60.8%]. Fifty-seven patients presented with LVEF ≤ 40% (heart failure with reduced ejection fraction (HFrEF)) and 244 with LVEF > 40%. In the multivariable analysis, N-terminal pro-B-type natriuretic peptide [NTproBNP] [*p* < 0.001, OR 1.000, 95%CI 1.000–1.000], baseline MLR [*p* < 0.020, OR 7.393, 95%CI 1.363–40.091] and female sex [*p* < 0.001, OR 0.308, 95%CI 0.160–0.593] were revealed as significant predictors of HFrEF. Baseline MLR weakly correlated with EuroScore II [Spearman r = 0.141, *p* = 0.015] and NTproBNP [r = 0.142, *p* = 0.014]. Cut-off values were established as 0.36 for MLR and 3927 pg/mL for NTproBNP. After excluding 147 patients with CAD, there was still a statistically significant difference in MLR between the subgroups [*p* = 0.024]. **Conclusions**: Increased values of MLR and NTproBNP together with female sex are predictive parameters for LVEF ≤ 40% in patients with severe aortic stenosis.

## 1. Introduction

Calcific aortic stenosis is the most common valvular heart disease, and its prevalence increases with age. It develops slowly with the progression of fibrosis, inflammation, oxidative stress, neovascularisation, and calcification of the valve and left ventricular myocardial tissues [1]. The pressure overload is compensated by left ventricular hypertrophy. With disease progression, worsening of myocardial function, impaired coronary flow reserve, and decrease in contractility occur [2]. In addition, concomitant coronary artery disease may affect left ventricular function. Thus, heart damage in aortic stenosis may result primarily from valvular defects or myocardial fibrosis [3]. The degree of left ventricular dysfunction was precisely defined in the latest guidelines [4] based on the left ventricular ejection fraction [LVEF], symptoms and signs, and objective evidence of cardiac abnormalities, with differentiation to heart failure with reduced ejection fraction [HFrEF, LVEF ≤ 40%], mildly reduced ejection fraction [HFmrEF, LVEF 41–49%] and preserved ejection fraction [HFpEF, LVEF ≥ 50%]. Currently, surgical aortic valve replacement [SAVR] and transcatheter aortic valve replacement [TAVI] are the only effective methods of treatment in patients with severe aortic stenosis [5]. Patients with co-morbidities and HFrEF are more often qualified for TAVI than surgical aortic valve replacement [SAVR] due to higher peri-operative risk [6]. Clinical and diagnostic assessment is crucial for post-procedural outcomes [7] and enables proper patients’ selection. Key research areas in TAVI include prosthesis durability, paravalvular leak, coronary access, conduction disorders, and stroke risk [8]. Improvement in left ventricular systolic function is one of the main goals as a clinical outcome.

Impaired left ventricular systolic function is associated with worse survival in the setting of the disease and worse post-procedural outcomes, both after SAVR and TAVI [9,10]. In daily practice, approximately one-third of patients with aortic stenosis have any reduction in systolic function [11], and severe LV dysfunction may be documented in even 13% of patients qualified for TAVI [9]. Even though the peri-operative risk is significantly higher in patients with left ventricular dysfunction, which is reflected in higher EuroScore II, the analyses presenting outcomes point out substantial improvement in LVEF and survival if the surgical or transcatheter procedure was conducted [12]. Early improvement in left ventricular contractility has been associated with beneficial long-term outcomes after SAVR and TAVI [10,13,14,15]. Lack of LVEF improvement by 30 days has been related to a 3-fold increase in 1-year mortality after TAVI [16]. The less invasive manner of TAVI and the lack of harmful effects of cardiopulmonary bypass were suggested as beneficial [6].

Several imaging tools are used for the assessment of heart failure, including transthoracic echocardiography [TTE] [6,17], lung ultrasonography [16], and cardiac magnetic resonance [CMR] [11]. Evaluation of the severity of aortic stenosis may be challenging in patients with severely reduced LVEF. Therefore, additional modalities should be implemented, such as computed tomography with evaluation of aortic valve calcification [18].

Inflammatory response is an important factor in the development and progression of aortic stenosis. It is also a hallmark pattern in advancing heart failure. Several biomarkers are used to facilitate diagnosis, assess disease progression, and respond to specific therapeutic approaches [19]. The role of immune ratios has been underlined in diagnosing and grading of several cardiovascular [20,21,22,23] and non-cardiovascular [24,25,26] disorders. Our previous study showed the association between parathyroid hormone concentration and inflammatory ratios, pointing out the role of activated inflammatory response in aortic stenosis [27]. Whole blood analysis enables simple and cost-effective evaluation of the inflammatory background of the disease. However, the practical implications of blood immune cell indices in aortic stenosis with systolic dysfunction are less known.

The aim of the study was to reveal demographic, clinical, and laboratory parameters that may differentiate patients with and without HFrEF associated with severe aortic stenosis. The hypothesis concerned immune ratios, representing typical inflammatory response in aortic stenosis and heart failure, which may serve as a beneficial, non-invasive, and easily obtained tool for HFrEF prediction.

## 2. Materials and Methods

This was a retrospective, observational, single-center study of all consecutive patients diagnosed with severe calcific aortic stenosis with overt heart failure qualified for TAVI at the structural heart-team center in the Department of Cardiology and Cardiac Surgery and Transplantology Department, Poznan University of Medical Sciences, between January 2013 and July 2019.

The heart team consisting of cardiologists [interventional and imaging specialists], cardiac surgeons, and cardiac anesthetists analyzed patients’ clinical profiles and qualified for SAVR, TAVI, or medical or palliative therapy. TAVI procedural planning included a clinical interview, physical examinations, laboratory assessments of blood samples, transthoracic echocardiography [TTE], computed tomography, and coronary angiography. More profound diagnostics and other specialistic consultations were adopted in case of significant co-morbidities or clinical uncertainties.

Inclusion criteria were as follows: 1. presence of severe aortic stenosis with heart failure symptoms; and 2. qualification for TAVI.

The key exclusion criterion was an active disease that could significantly influence the immune ratios. All patients’ co-morbidities were meticulously investigated and reported. Exclusion criteria were 1. patients with hematological and neoplastic diseases; and 2. patients with a diagnosis of cardiac amyloidosis. Moreover, we did not include a cohort of patients using sacubitril-valsartan and flosins to avoid in order to avoid any potential bias related to introducing newer therapies in heart failure.

Demographic [sex, age] and clinical [body mass index [BMI], arterial hypertension [HA], atrial fibrillation [AF], diabetes mellitus [DM], coronary artery disease [CAD], previous interventions including percutaneous coronary intervention [PCI] and coronary artery bypass grafting [CABG], chronic obstructive pulmonary disease [COPD], chronic kidney disease with glomerular filtration rate [GFR] below 59 mL/min/1.73 m^2^, data were analyzed. The diagnosis of HA, DM and COPD were documented based on the patient’s history, medical records, and medication. CAD was assessed based on coronary angiography or previous coronary angiogram reports. If necessary, PCI was performed. AF was documented in an electrocardiogram (ECG) or based on the patient’s history and medication.

Transthoracic echocardiography [TTE] was performed on each patient by a team of experienced echocardiographers according to general guidelines on valvular disease management [5]. The TTE included an assessment of aortic stenosis severity based on the peak and mean transvalvular gradients, aortic valve area [AVA], and left ventricular performance with LVEF and left ventricular hypertrophy. Baseline blood samples were collected at admission for the simple whole blood analysis, N-terminal pro-B-type natriuretic peptide [NTproBNP], and serum creatinine. The glomerular filtration rate was calculated using the Modification of Diet in Renal Disease [MDRD] Study equation for kidney function assessment and grading. Inflammatory ratios were calculated based on the whole blood count analysis, including neutrophil-to-lymphocyte ratio [NLR], monocyte-to-lymphocyte ratio [MLR], and platelet-to-lymphocyte ratio [PLR].

The heart failure was diagnosed, and the grade was described based on the current guidelines on its diagnosis and management [5]. Patients’ symptoms of heart failure were categorized based on New York Heart Association [NYHA] classification. The procedural risk profile was assessed using EuroScore II.

Outcomes of interest included differences in patients’ characteristics and laboratory results stratified by clinical and echocardiographic assessment of heart failure classification.

The study group was divided based on an echocardiographic assessment of LVEF into group 1 with LVEF ≤ 40% and group 2 with heart failure with LVEF > 40% [mildly reduced ejection fraction [HFmrEF] and preserved ejection fraction [HFpEF]].

The study was approved by the Institutional Ethics Committee [No 272/2021 dated 8 April 2021] and respected the principles outlined in the Declaration of Helsinki. Patients provided written informed consent to participate in the study. The STROBE guidelines were followed.

### Statistical Analysis

The Shapiro–Wilk test was used to assess the data distribution. Continuous variables were presented as mean and standard deviation [SD], or median and interquartile range [Q1–Q3], if normally or not normally distributed data were presented, respectively. Student t-test or non-parametric Mann–Whitney test or ANOVA test were used where applicable. Categorical data were expressed as numbers and percentages and compared with Fisher’s exact test. Univariable and multivariable analyses included demographic, clinical, and laboratory data to reveal predictors of HFrEF in patients with severe aortic stenosis in logistic regression with a backward selection method. Spearman correlation analysis was used to describe the correlation between the variables. Statistical analysis was performed using JASP software [JASP Team; 2020. Version 0.13.1]. The cut-off value was established using the Youden index in PQStat software [Version 1.8.6]. *p* < 0.05 was considered statistically significant.

## 3. Results

### 3.1. Patients Characteristics

**Figure 1:**
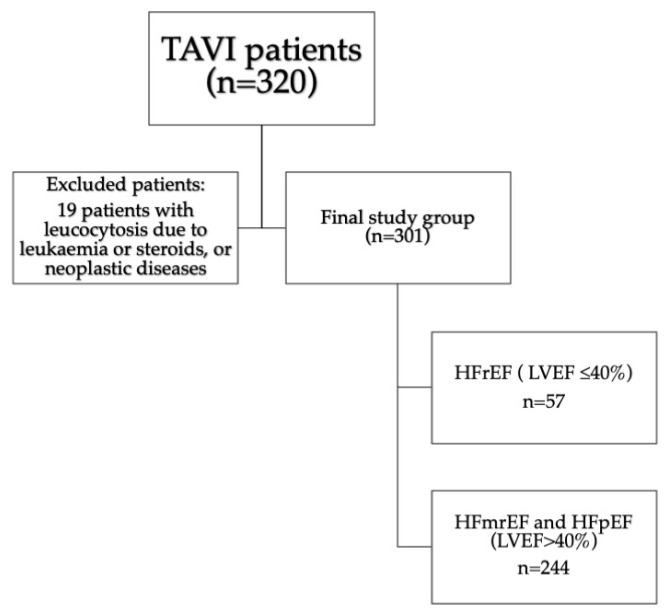
Study flow-chart. Abbreviations: HFmrEF—heart failure with mildly reduced ejection fraction, HFpEF—heart failure with preserved ejection fraction, HFrEF—heart failure with reduced ejection fraction, LVEF—left ventricular ejection fraction, TAVI—transcatheter aortic valve implantation.

We analyzed 320 patients with severe aortic stenosis presenting with heart failure who were qualified for TAVI. Nineteen patients were excluded due to the presence of exclusion criteria. The final study population comprised 301 consecutive patients [133 males [44%]; median [Q1–3] age of 80 [75–83] years]. All patients manifested overt heart failure in the New York Heart Association [NYHA] classification of II to IV. Median [Q1–3] aortic peak transvalvular gradient was 89 [78–105] mmHg. Among them, 147 [48.8%] presented coronary artery disease, with significant stenosis treated with PCI during the qualification process or in the past, or previous history of CABG. Fifty-seven [18.9%] patients presented with LVEF ≤ 40%, comprising group 1, and 244 patients with LVEF over 40% [group 2]. The study flowchart with inclusion and exclusion criteria is presented in Figure 1.

Co-morbidities included arterial hypertension [n = 227, 75.4%], diabetes mellitus [n = 124, 41.2%], chronic obstructive pulmonary disease [n = 51, 16.9%], atrial fibrillation [n = 118, 39.2%], chronic kidney disease defined as GFR < 59 mL/min/1.73 m^2^ [n = 183, 60.8%].

A comparison of demographic and clinical data between subgroups is presented in Table 1 and Supplementary Materials.

Blood samples were collected at baseline and compared between subgroups (Table 2).

### 3.2. Uni and Multivariable Analysis

**Figure 2:**
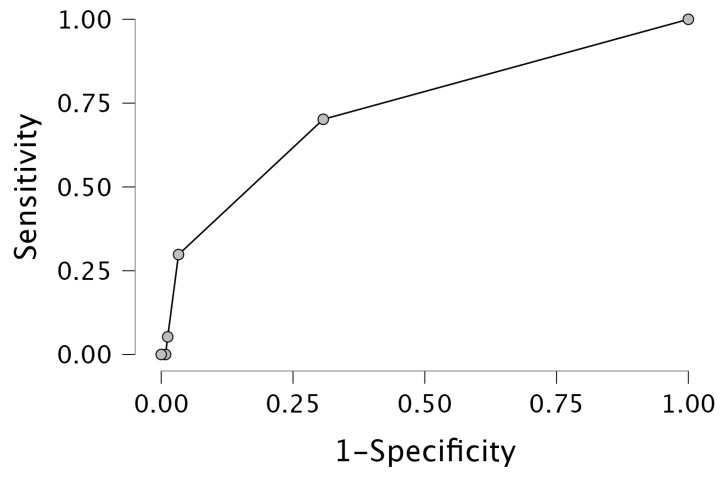
Receiver operating characteristic [ROC] curve for HFrEF prediction.

In the multivariable analysis (Table 3), NTproBNP [*p* < 0.001, OR 1.000, 95%CI 1.000–1.000], baseline MLR [*p* < 0.020, OR 7.393, 95%CI 1.363–40.091] and female sex [*p* < 0.001, OR 0.308, 95%CI 0.160–0.593] were revealed as significant predictors of HFrEF, with an AUC of 0.756. The ROC analysis is presented in Figure 2.

### 3.3. MLR and NTproBNP Comparisons between Subgroups (Figure 3)

Baseline MLR weakly correlated with EuroScore II [Spearman r = 0.141, *p* = 0.015] and with NtproBNP [Spearman r = 0.142, *p* = 0.014].

Cut-off values were established as 0.36 with AUC 0.624, 54% sensitivity and 69.7% specificity, and 3927 pg/mL. with AUC 0.753, 73.7% sensitivity and 72.5% specificity, for MLR and for NTproBNP, respectively.

### 3.4. Sub-Analysis

**Figure 4:**
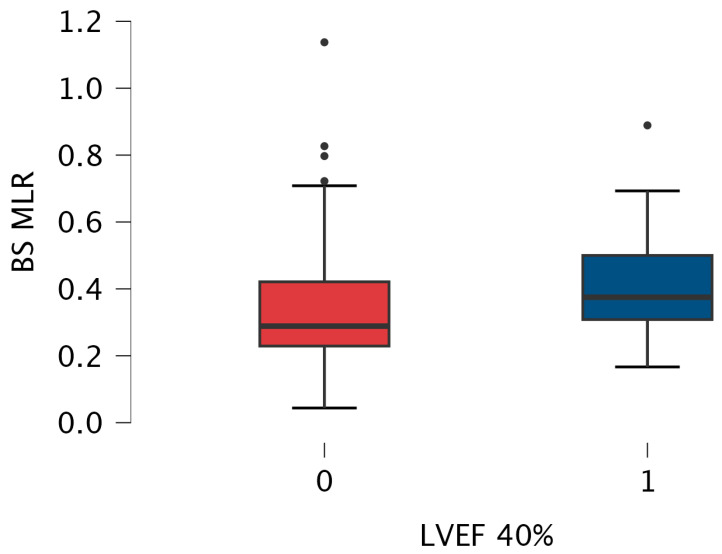
Box-plot for baseline MLR values measured in patients with LVEF > 40% (0) and below 40% (1) without coronary artery disease. The box-plot shows the minimum and maximum values [whiskers], median [black line] and interquartile range [box] values for each variable. Outliers are shown with black points. HFrEF patients had significantly higher MLR than patients with LVEF > 40%. Abbreviations: BS—baseline, LVEF—left ventricular ejection fraction, MLR—monocyte-lymphocyte ratio.

Since both subgroups differed in coronary artery disease prevalence, we performed a sub-analysis by excluding those 147 patients. In the remaining group, there was still a statistically significant difference in MLR value between patients with LVEF ≤ 40% and >40% (median [Q1–Q3] 0.375 [0.308–0.500] vs. 0.289 [0.229–0.421], respectively, *p* = 0.024) (Figure 4).

## 4. Discussion

The main findings of our study are the predictive significance of MLR, NTproBNP, and female sex for the prevalence of HFrEF in patients with severe aortic stenosis. To our knowledge, this is the first report presenting the usefulness of MLR value in differentiating the HFrEF type.

The measurement of NTproBNP, according to current guidelines, together with echocardiography, enables clinicians to define subtypes of HF. Inflammatory biomarkers are gaining increasing attention since their usefulness was shown in several studies on coronary artery disease, carotid atherosclerosis, heart failure, or myocardial damage [22,28,29]. A low-grade, enduring inflammatory process is implicated in the background of cardiovascular diseases, including coronary artery disease and aortic stenosis.

MLR is a widely available laboratory measurement that is easily accessible and of low cost and may reflect the pathophysiological effects of inflammatory response. Hua et al. [30] investigated overall and cardiovascular mortality in the general population and showed significant differences in MLR values between survivors and non-survivors. Individuals in the highest tertile of MLR were higher risk of mortality. In the meta-analysis of nineteen articles by Vakhshoori et al. [31], higher MLR was associated with an increased risk of major adverse events [MACE] in patients with coronary artery disease. The same scientific group analyzed the current literature to establish the role of MLR or LMR and considered these ratios as the best prognostic tools for mortality risk assessment in heart failure [32].

According to our results, MLR may be beneficial in daily routine practice in tailored risk stratification for the degree of heart failure advancement in patients with aortic stenosis. Patients with HFrEF and aortic stenosis, and particularly those with low flow, low gradient type, are among the most challenging ones concerning both diagnosis and treatment. Left ventricular dysfunction related to myocardial infarction is the most studied etiology of heart failure [33]. In our study, both subgroups with concomitant coronary artery disease and pure aortic stenosis presented with significantly higher MLR values in HFrEF patients.

Though older studies described aortic stenosis mainly as a degenerative disease, current reports underline its inflammatory background. Aortic stenosis is determined by progressive inflammatory and fibrocalcific processes. Mueller et al. [34] showed the key role of macrophage migration inhibitory factor [MIF] and its interplay with circulating and valve resident monocytes in thrombo-inflammation during disease progression. Immune cells in the innate and adaptive models play an important role in the progression of atherosclerosis [35]. Circulating monocytes and resident vascular macrophages are recruited in response to various inflammatory stimuli and subsequently differentiate into macrophages and foam cells [36]. The activation is reflected in increased absolute or relative monocyte count in blood [30]. Monocytes are capable of producing and releasing various inflammatory factors, such as cytokines and chemokines, reactive oxidative substances, and metalloproteinases [37,38]. They contribute to atherosclerotic plaque initiation and progression in coronary arteries [39], carotid arteries [38], and aortic atherosclerosis [40]. The monocyte subtypes may have different potential for adhesion and migration and altered entry to the atherosclerotic plaque [41]. Han et al. [42] found that monocyte count was positively associated with the rapid progression of aortic stenosis. Post-procedural monocytosis between 1 and 3 days after TAVI was associated with 30-day MACE [composite of stroke, acute myocardial infarction, and death] in Navani et al. [43] analysis. This may suggest that minimizing peri-procedural inflammation may improve outcomes.

Coronary artery disease and aortic stenosis share several pathophysiological mechanisms and risk factors [44]. Patients with co-existence of both diseases present a larger number of comorbidities compared to those with isolated disorders [42]. The optimal timing of percutaneous coronary intervention in TAVI patients is still the reason for many debates [45]. On the contrary, patients with similar profile morbidity and risk factors may present with pure aortic stenosis or coronary artery disease. Thus, pathomechanisms of their concomitant or isolated development need further investigation.

Several aforementioned studies proved the inflammatory background of aortic stenosis development and progression and underlined the significance of monocytes in these processes. Our study, by analysis of simple laboratory and clinical variables, confirmed the complexity of inflammatory response in aortic stenosis pathophysiology. Heart failure occurrence, and particularly marked left ventricular dysfunction, is associated with monocyte count and MLR increase, pointing out the clinical implication of monocytes for aortic stenosis advancement. These observations are important evidence for searching for new forms of therapy based on minimizing inflammation, both at the early and late stages of the disease. In the future, they might be a background for any anti-inflammatory therapy directed to alleviation of immune system activation, particularly specific cell lines. Since aortic stenosis triggers left ventricular hypertrophy and myocardial fibrosis [46], it causes irreversible damage in the myocardial wall, remaining to a considerable extent even after successful interventional treatment. Fibrosis development in tissues is also considered a process of reparation associated with increased activity of some pro-inflammatory cells. Thus, early recognition of left ventricular systolic dysfunction is an important issue in patients’ evaluation. Based on our study, we suggest that simple whole blood analysis with monocyte count and MLR monitoring may be beneficial in daily ambulatory clinics, and increased MLR values may indicate the necessity for broadening the diagnostics with imaging studies.

### Limitations

Valsartan/sacubitril and SGLT2 inhibitors were introduced to the current guidelines as the golden standard for the management of heart failure. However, the gross part of our study group was recruited before the recommendation of SGLT2 inhibitors. Therefore, we did not include patients using these medications in our analysis to avoid bias related to the direct effect of this therapy and difficulties in assessing patients with and without them. However, we believe that an additional analysis of the influence of SGLT2 inhibitors on the MLR level and its predictive value for HFrEF would be beneficial. Further analyses in larger populations are necessary.

## 5. Conclusions

Increased values of MLR and NTproBNP together with the female sex are predictive parameters for reduced ejection fraction in patients with severe aortic stenosis. MLR, as an easily available laboratory measurement, may be a useful tool in heart failure patients.

## Figures and Tables

**Figure 3:**
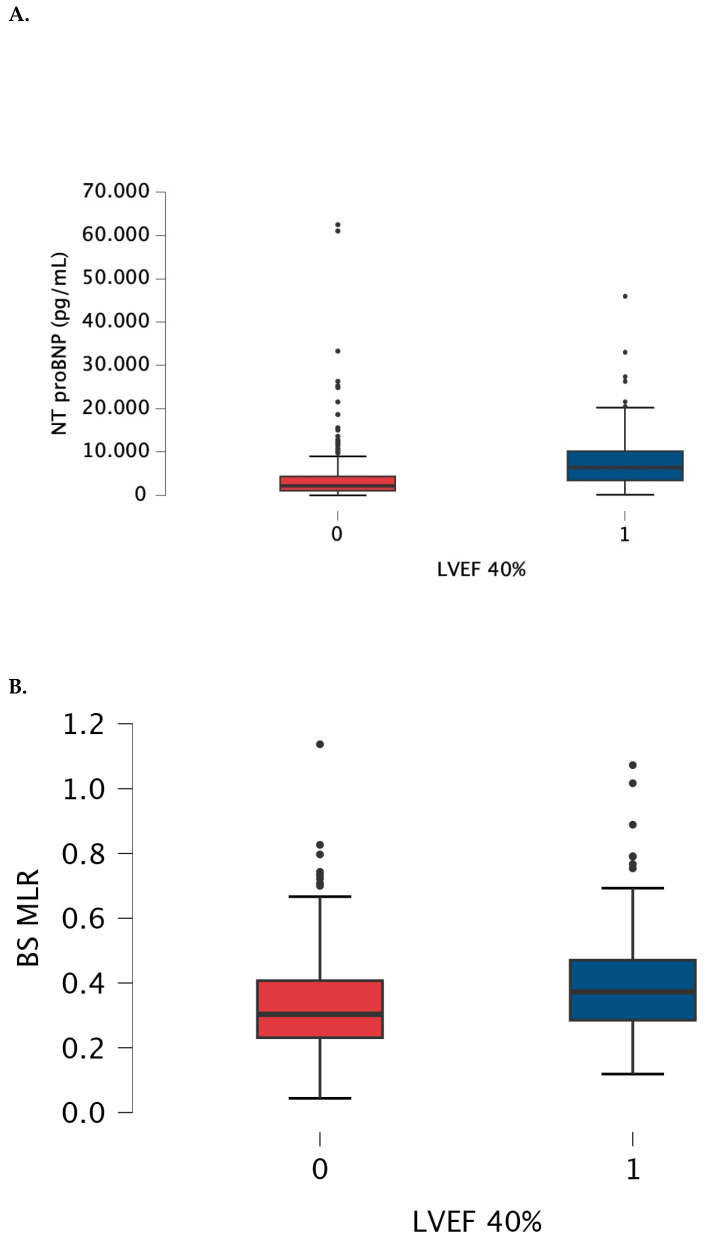
Box-plot for NRproBNP (**A**) and baseline MLR (**B**) values measured in patients with LVEF > 40% (0) and below 40% (1). The box-plot shows the minimum and maximum values [whiskers], median [black line] and interquartile range [box] values for each variable. Outliers are shown with black points. HFrEF patients had significantly higher NTproBNp and MLR than patients with LVEF > 40%. Abbreviations: BS—baseline, LVEF—left ventricular ejection fraction, NTproBNP—N-terminal pro-B-type natriuretic peptide, MLR—monocyte-lymphocyte ratio.

**Table 1:** Demographic and clinical data.

	Group 1 LVEF ≤ 40% n = 57	Group 2 LVEF > 40% n = 244	p Value
Age [years] [median, Q1–Q3]	78 [71–82]	80 [76–83]	0.013
Female sex [n, %] BMI [median, Q1–Q3]	19 [33] 26.8 [22.8–28.7]	149 [61.1] 27.2 [24.3–30.5]	<0.001 0.097
EuroScore II [median, Q1–Q3]	8.34 [5.77–16.11]	4.57 [2.87–7.75]	0.001
DM [n, %]	29 [50.9]	95 [38.9]	0.103
HA [n, %]	38 [66.7]	189 [77.5]	0.123
COPD [n, %]	11 [19.3]	40 [16.4]	0.563
AF [n, %]	20 [35.1]	98 [40.2]	0.548
Coronary artery disease [n, %]	36 [63.2]	111 [45.5]	0.019
Previous myocardial infarction [n, %]	31 [54.4]	71 [29.1]	<0.001
Previous PCI [n, %]	13 [50]	53 [42.7]	0.522
Previous CABG [n, %]	19 [33.3]	41 [16.8]	0.009
Previous stroke or TIA [n, %]	8 [14]	46 [18.9]	0.449
NYHA III–IV [n, %]	54 [94.7]	215 [88.1]	0.230
Mean transvalvular gradient [mmHg] [median, Q1–Q3]	41 [35–52]	58 [49–68]	<0.001
Peak transvalvular gradient [mmHg] [median, Q1–Q3]	70 [58–86]	92 [81.5–109.1]	<0.001
LVEF [%] [median, Q1–Q3]	35 [25–40]	60 [50–60]	<0.001
PASP [mmHg] [median, Q1–Q3]	46 [40–60]	42 [35–50]	0.003
AVA [cm2] [median, Q1–Q3]	0.7 [0.6–0.78]	0.6 [0.5–0.7]	0.193

Abbreviations: AF—atrial fibrillation, AVA—aortic valve area, BMI—body mass index, CABG—coronary artery bypass grafting, COPD—Chronic obstructive pulmonary disease, DM—diabetes mellitus, HA—arterial hypertension, LVEF—left ventricular ejection fraction, NYHA—New York Heart Association classification, Q—quartile, PASP—pulmonary artery systolic pressure, PCI—percutaneous coronary intervention, TIA—transient ischemic attack.

**Table 2:** Laboratory results.

	Group 1 LVEF ≤ 40% n = 57	Group 2 LVEF > 40% n = 244	*p* Value
NTproBNP pg/mL	6425 [3434–10,200]	2080 [1015–4357.8]	<0.001
GFR [ml/min/1.73 m^2^] [median, Q1–Q3]	55 [38–66]	55 [45–67]	0.299
Neu [10 × 9/L] [median, Q1–Q3]	4.94 [4.31–6.15]	4.7 [3.67–5.93]	0.157
Lymp [10 × 9/L] [median, Q1–Q3]	1.48 [1.05–1.76]	1.39 [1.08–1.82]	0.736
Mono [10 × 9/L] [median, Q1–Q3]	0.52 [0.36–0.65]	0.41 [0.33–0.53]	0.003
MLR [median, Q1–Q3]	0.37 [0.29–0.47]	0.30 [0.23–0.41]	0.004
NLR [median, Q1–Q3]	3.68 [2.77–4.93]	3.39 [2.39–4.54]	0.132
PLR [median, Q1–Q3]	109 [99–123.9]	128.6 [98.2–194.6]	0.521
Hemoglobin [mmol/L] [median, Q1–Q3]	8.1 [7.6–8.9]	7.9 [7.2–8.5]	0.053

Abbreviations: NTproBNP—N-terminal pro-B-type natriuretic peptide, GFR—glomerular filtration rate, Lymp—lymphocyte count, Mono—monocyte count, MLR—monocyte-to-lymphocyte ratio, Neu—neutrophil count, NLR—neutrophil-to-lymphocyte ratio, PLR—platelet-to-lymphocyte ratio, Q–quartile.

**Table 3:** Uni and multivariable analysis of patients with HFrEF and HFmrEF/HFpEF.

	Univariable Analysis	*p*	Multivariable Analysis	*p*
Age	OR 0.95 [95%CI 0.91–0.99]	0.014		
Female sex	OR 0.32 [95%CI 0.17–0.59]	<0.001	OR 0.308, 95%CI 0.160–0.593	<0.001
BMI	OR 0.94 [95%CI 0.88–1.01]	0.079		
AF	OR 0.81 [95%CI 0.44–1.47]	0.48		
Coronary artery disease	OR 2.05 [95%CI 1.13–3.72]	0.018		
Previous myocardial infarction	OR 2.9 [95%CI 1.61–5.24]	<0.001		
Previous CABG	OR 2.48 [95%CI 1.30–4.72]	0.006		
HA	OR 0.32 [95%CI 0.31–1.09]	0.091		
DM	OR 1.62 [95%CI 0.91–2.90]	0.101		
COPD	OR 1.22 [95%CI 0.58–2.56]	0.60		
NTproBNP	OR 1.00 [95%CI 1.00–1.00]	<0.001	OR 1.000, 95%CI 1.000–1.000	<0.001
GFR	OR 0.99 [95%CI 0.98–1.01]	0.26		
NLR	OR 1.08 [95%CI 0.95–1.22]	0.25		
MLR	OR 14.35 [95%CI 2.88–71.45]	0.001	OR 7.393, 95%CI 1.363–40.091	0.020
PLR	OR 1.00 [0.99–1.01]	0.47		

Abbreviations: AF—atrial fibrillation, BMI—body mass index, CABG—coronary artery bypass grafting, COPD—Chronic obstructive pulmonary disease, DM—diabetes mellitus, GFR—glomerular filtration rate, HA—arterial hypertension, MLR—monocyte-to-lymphocyte ratio, NLR—neutrophil-to-lymphocyte ratio, NTproBNP—N-terminal pro-B-type natriuretic peptide, PLR—platelet-to-lymphocyte ratio.

## Data Availability

All data will be available for 3 years following the publication after a reasonable request is presented by e-mail correspondence to the corresponding author.

## Supplementary Materials

The following supporting information can be downloaded at: https://www.mdpi.com/article/10.3390/jcm13206249/s1, Table S1: STROBE Statement—checklist of items that should be included in reports of observational studies; Table S2: Demographic and clinical data in patients divided according to LVEF to HFrEF (LVEF≤40%), HFmrEF (LVEF 41–49)% and HFpEF (LVEF≥50%)
