# Supplementary material for: Predictive Value of Monocyte-To-Lymphocyte Ratio in Differentiating Heart Failure with Reduced Ejection Fraction in Patients with Severe Aortic Stenosis—A Retrospective Analysis
Source: J Clin Med. 2024 Oct 19;13(20):6249. doi: 10.3390/jcm13206249 (PMC11508807; doi:10.3390/jcm13206249)
Supplement: Supplementary file 1 [file jcm-13-06249-s001.zip › jcm-3230005-supplementary.pdf]

Suppl. Table.

Table S1. STROBE Statement—checklist of items that should be included in reports of observational studies

	Item No.	Recommendation	Page No.	Relevant text from manuscript
**Title and abstract**	1	(*a*) Indicate the study's design with a commonly used term in the title or the abstract	1	"Predictive Value of Monocyte-to-Lymphocyte Ratio in Differentiating Heart Failure with Reduced Ejection Fraction in Patients with Severe Aortic Stenosis – A Retrospective Analysis" Study design related to prediction of heart failure (HF) with reduced ejection fraction (EF) (HFrEF) in patients with severe aortic stenosis with use of clinical and laboratory variables. The study was performed in the retrospective manner based on the patients population treated with TAVI in Poznan cardiac-surgical centre
		(*b*) Provide in the abstract an informative and balanced summary of what was done and what was found	1	The abstract provided a short introduction to the topic of heart failure in patients with severe aortic stenosis. The analysis of clinical and laboratory parameters as predictors of HFrEF in aortic stenosis patients was the aim of the study. The summary of results and a conclusion was presented.
**Introduction**				
Background/rationale	2	Explain the scientific background and rationale for the investigation being reported	1-2	Presentation of aortic stenosis and difficulties related to left ventricular systolic dysfunction was described.

				Diagnostic challenges related to impaired left ventricular dysfunction were pointed out. The role on inflammatory response in cardiological disease and significance of immune ratios were described.
Objectives	3	State specific objectives, including any prespecified hypotheses	2	The diagnostic significance of immune ratios in aortic stenosis with HFrEF is unknown.<br>The aim of the study was to reveal demographic, clinical, and laboratory parameters that may differentiate patients with and without HFrEF associated with severe aortic stenosis.<br>The hypothesis concerned immune ratios, representing typical inflammatory response in aortic stenosis and heart failure, which may serve as a beneficial, non-invasive, and easily obtained tool for HFrEF prediction.
**Methods**				
Study design	4	Present key elements of study design early in the paper	2-3	Study project included patients with severe symptomatic aortic stenosis presenting with heart failure who were diagnosed by TAVI protocol and qualified for TAVI. The Methods section provides a detailed information on methods of clinical and imaging diagnosis. Laboratory examinations were explained. All patients underwent echocardiographic evaluation with aortic

				stenosis assessment AND left ventricular ejection fraction assessment. Based on the results, the study group was divided according to an echocardiographic assessment of LVEF into group 1 with LVEF <40% and group 2 with heart failure with LVEF>40% [mildly reduced ejection fraction [HFmrEF] and preserved ejection fraction [HFpEF]].
Setting	5	Describe the setting, locations, and relevant dates, including periods of recruitment, exposure, follow-up, and data collection	2	Patients were diagnosed and treated in cardiological-cardiosurgical centre in Poznan University of Medical Sciences, Poznan, Poland between January 2013 and July 2019. Follow-up was conducted, however was not presented in the current study, as it was not a topic of the analysis. Data collection modes were explained.
Participants	6	(*a*) *Cohort study*—Give the eligibility criteria, and the sources and methods of selection of participants. Describe methods of follow-up<br>*Case-control study*—Give the eligibility criteria, and the sources and methods of case ascertainment and control selection. Give the rationale for the choice of cases and controls<br>*Cross-sectional study*—Give the eligibility criteria, and the sources and methods of selection of participants	2-3	All patients qualified for TAVI in the analysed period were assessed to present with inclusion criteria or were excluded based on the exclusion data.<br>Inclusion criteria (1. presence of severe aortic stenosis with heart failure symptoms and 2. qualification for TAVI.) and exclusion criteria (The key exclusion criterion was an active disease that could significantly influence the immune ratios. All patients' co-morbidities were meticulously investigated and reported. Exclusion

				criteria were 1. patients with hematological and neoplastic diseases, 2., patients with a diagnosis of cardiac amyloidosis. Moreover, we did not include a cohort of patients using sacubitril-valsartan and flosins to avoid in order to avoid any potential bias related to introducing newer therapies in heart failure) were described
		(*b*) *Cohort study*—For matched studies, give matching criteria and number of exposed and unexposed<br>*Case-control study*—For matched studies, give matching criteria and the number of controls per case	-	Study subgroups were not matched.
Variables	7	Clearly define all outcomes, exposures, predictors, potential confounders, and effect modifiers. Give diagnostic criteria, if applicable	3	Variables addressing aortic stenosis, heart failure and co-morbidities were defined. Examinations which were performed were stated. The explanation of calculation of immune ratios were given.<br>The study group was divided based on an echocardiographic assessment of LVEF into group 1 with LVEF 40% and group 2 with heart failure with LVEF>40% [mildly reduced ejection fraction [HFmrEF] and preserved ejection fraction [HFpEF]].
Data sources/ measurement	8*	For each variable of interest, give sources of data and details of methods of assessment (measurement). Describe comparability of assessment methods if there is more than one group	*2-3*	Information on diagnosis of aortic stenosis, heart failure and co-morbidities.<br>Both sub-groups were compared by the same variables occurrence.
Bias	9	Describe any efforts to address potential sources of bias	3	The same pharmacotherapy was implemented in the study group. Since

				the study group comprised patients at different years, patients with newest medication (V/S and flosins) were excluded to avoid the risk of bias related to difference in treatment startegies.
Study size	10	Explain how the study size was arrived at	2	This is an all-comers study.

Continued on next page

Quantitative variables	11	Explain how quantitative variables were handled in the analyses. If applicable, describe which groupings were chosen and why	2-3	Laboratory results, operative risk scores were presented as quantitative variables and compared between subgroups
Statistical methods	12	(*a*) Describe all statistical methods, including those used to control for confounding	3	All statistical analyses used in the study were presented in the statistical analysis section
		(*b*) Describe any methods used to examine subgroups and interactions	3	Student t-test or non-parametric Mann-Whitney test or ANOVA test were used where applicable to compare the subgroups. Categorical data were expressed as numbers and percentages and compared with Fisher exact test.
		(*c*) Explain how missing data were addressed	NA	We used detailed clinical and laboratory data.
		(*d*) *Cohort study*—If applicable, explain how loss to follow-up was addressed<br>*Case-control study*—If applicable, explain how matching of cases and controls was addressed<br>*Cross-sectional study*—If applicable, describe analytical methods taking account of sampling strategy	NA	All-comers strategy
		(*e*) Describe any sensitivity analyses	NA	
**Results**				
Participants	13*	(a) Report numbers of individuals at each stage of study—eg numbers potentially eligible, examined for eligibility, confirmed eligible, included in the study, completing follow-up, and analysed	4, Fig1	We analyzed 320 patients with severe aortic stenosis presenting with heart failure who were qualified for TAVI. Nineteen patients were excluded due to the presence of exclusion criteria. The final study population comprised 301 consecutive patients [133 males [44%]; median [Q1-3] age of 80 [75-83] years].
		(b) Give reasons for non-participation at each stage	4, Fig 1	We presented 19 patients with exclusion criteria.
		(c) Consider use of a flow diagram	Fig 1	We presented study flowchart

Descriptive data	14*	(a) Give characteristics of study participants (eg demographic, clinical, social) and information on exposures and potential confounders	4-6	Presentation of demographics, clinical presentation of aortic stenosis and heart failure, and co-morbidities were described.
		(b) Indicate number of participants with missing data for each variable of interest		NA
		(c) *Cohort study*—Summarise follow-up time (eg, average and total amount)		-
Outcome data	15*	*Cohort study*—Report numbers of outcome events or summary measures over time		-
		Case-control study—Report numbers in each exposure category, or summary measures of exposure		-
		Cross-sectional study—Report numbers of outcome events or summary measures	*4*	*Fifty-seven [18.9%] patients presented with LVEF<40%, comprising group 1, and 244 patients with LVEF over 40% [group 2].*
Main results	16	(*a*) Give unadjusted estimates and, if applicable, confounder-adjusted estimates and their precision (eg, 95% confidence interval). Make clear which confounders were adjusted for and why they were included	7-10	Uni and multivariable analyses were shown.
		(*b*) Report category boundaries when continuous variables were categorized		NA
		(*c*) If relevant, consider translating estimates of relative risk into absolute risk for a meaningful time period		NA

Continued on next page

Other analyses	17	Report other analyses done—eg analyses of subgroups and interactions, and sensitivity analyses	10	Sub-analysis without CAD patients was presented.
**Discussion**				
Key results	18	Summarise key results with reference to study objectives	11	The main findings of our study are the predictive significance of MLR, NTproBNP, and female sex for the prevalence of HFrEF in patients with severe aortic stenosis. To our knowledge, this is the first report presenting the usefulness of MLR value in differenti-ating the HFrEF type.
Limitations	19	Discuss limitations of the study, taking into account sources of potential bias or imprecision. Discuss both direction and magnitude of any potential bias	12	Valsartan/sacubitril and SGLT2 inhibitors have been introduced to the current guidelines as the golden standard for the management of heart failure. However, the gross part of our study group was recruited before the recommendation of SGLT2 in-hibitors. Therefore, we did not include patients using these medications in our analysis to avoid bias related to the direct effect of this therapy and difficulties in assessing patients with and without them. However, we believe that an additional analysis of the influence of SGLT2 inhibitors on the MLR level and its predictive value for HFrEF would be beneficial.
Interpretation	20	Give a cautious overall interpretation of results considering objectives, limitations, multiplicity of analyses, results from similar studies, and other relevant evidence	11-12	We discussed our results in comparison with previously published data
Generalisability	21	Discuss the generalisability (external validity) of the study results	12	we believe that an additional analysis of the influence of SGLT2 inhibitors on the MLR level and its predictive value for HFrEF would be beneficial. Further analyses in the larger populations are necessary.

Other information				
Funding	22	Give the source of funding and the role of the funders for the present study and, if applicable, for the original study on which the present article is based	12	No funding

*Give information separately for cases and controls in case-control studies and, if applicable, for exposed and unexposed groups in cohort and cross-sectional studies.

Note: An Explanation and Elaboration article discusses each checklist item and gives methodological background and published examples of transparent reporting. The STROBE checklist is best used in conjunction with this article (freely available on the Web sites of PLoS Medicine at http://www.plosmedicine.org/, Annals of Internal Medicine at http://www.annals.org/, and Epidemiology at http://www.epidem.com/). Information on the STROBE Initiative is available at www.strobe-statement.org.

Table S2. Demographic and clinical data in patients divided according to LVEF to HFrEF (LVEF≤40%), HFmrEF (LVEF 41-49)% and HFpEF (LVEF≥50%)

	Patients with HFrEF n=57	Patients with HFmrEF n=32	Patients with HFpEF n=212	p value HFmrEF vs HFpEF	p value all subgroups
Age [years] [median, Q1-Q3]	78 [71-82]	80 [75.8-83]	80 [76-83]	0.793	0.045
Female sex [n, %]	19 [33]	16 [50]	133 [62.7]	0.178	<0.001
BMI [median, Q1-Q3]	26.8 [22.8-28.7]	27 [23.9-30.5]	27.2 [24.3-30.5]	0.793	0.212
EuroScore II [median, Q1-Q3]	8.34 [5.77-16.11]	9.36 [5.27-12.9]	4.27 [2.69-7.27]	<0.001	<0.001
DM [n, %]	29 [50.9%]	17 [53.1]	78 [36.8]	0.084	0.056
HA [n, %]	38 [66.7%]	20 [62.6]	169 [79.7]	0.040	0.025
COPD [n, %]	11 [19.3]	4 [12.5]	36 [17]	0.618	0.714
AF [n, %]	20 [35.1]	15 [46.9]	83 [39.2]	0.442	0.550
Coronary artery disease [n, %]	36 [63.2]	17 [53.1]	94 [44.3]	0.447	0.036
Previous myocardial infarction [n, %]	31 [54.4]	16 [50]	55 [25.9]	0.011	<0.001
Previous PCI [n, %]	13 [50]	9 [43]	26 [26.3]	0.185	0.565
Previous CABG [n, %]	19 [33.3]	6 [18.8]	35 [33.3]	0.800	0.018
Previous stroke or TIA [n, %]	8 [14]	8 [25]	38 [17.9]	0.338	0.433
NYHA III-IV [n, %]	54 [94.7]	29 [90.6]	186 [87.7]	0.777	0.305

Mean transvalvular gradient [mmHg] [median, Q1-Q3]	41 [35-52]	52 [45-57]	59 [50-69]	0.009	<0.001
Peak transvalvular gradient [mmHg] [median, Q1-Q3]	70 [58-86]	83 [73-95.5]	93.4 [82.8-110.3]	0.002	<0.001
LVEF [%] [median, Q1-Q3]	35 [25-40]	45 [45-45]	60 [55-60]	<0.001	<0.001
PASP [mmHg] [median, Q1-Q3]	46 [40-60]	45 [30-50.5]	42 [35-50]	0.921	0.004
AVA [cm^2] [median, Q1-Q3]	0.7 [0.6-0.78]	0.7 [0.6-0.8]	0.6 [0.5-0.7]	0.298	0.235

Abbreviations: AF – atrial fibrillation, AVA – aortic valve area, BMI – body mass index, CABG - coronary artery bypass grafting, COPD - Chronic obstructive pulmonary disease, DM – diabetes mellitus, HA – arterial hypertension, HFpEF – heart failure with preserved ejection fraction, HFmrEF – heart failure with mildly reduced ejection fraction, HFrEF – heart failure with reduced ejection fraction, LVEF – left ventricular ejection fraction, NYHA – New York Heart Association classification, Q- quartile, PASP – pulmonary artery systolic pressure, PCI – percutaneous coronary intervention, TIA – transient ischemic attack.
